# Solvatochromic and Computational Study of Three Benzo-[f]-Quinolinium Methylids with Photoinduced Charge Transfer

**DOI:** 10.3390/molecules30153162

**Published:** 2025-07-29

**Authors:** Mihaela Iuliana Avadanei, Ovidiu Gabriel Avadanei, Dana Ortansa Dorohoi

**Affiliations:** 1 Petru Poni Institute of Macromolecular Chemistry, 41A Gr. Ghica Voda Alley, 700487 Iasi, Romania; 2 Faculty of Physics, Alexandru Ioan Cuza University, 11 Carol I Blvd, 700506 Iasi, Romania; minu@uaic.ro

**Keywords:** negative solvatochromism, benzo-f-quinolinium methylids, charge transfer, intermolecular interactions, excited state dipole moment

## Abstract

The solvatochromic properties of 48 solvents of three benzo-[f]-quinolinium methylids (BfQs) were analyzed within the theories of the variational model and Abe’s model of the liquid. The electro-optical properties of BfQs in the first excited state were determined based on the charge transfer process that occurs from the ylid carbon to the nitrogen atom. The dipole moments and the polarizabilities in the first excited state were calculated according to the two models. The quantum chemical calculations helped in understanding the relationship between the molecular structure and absorption properties of the ground state. It is concluded that several key parameters modulate the strength of the charge transfer and they work in synergy, and the most important are as follows: (i) isomerism around the single polar bond, and (ii) the properties of the solvent. The link between geometrical conformation and the zwitterionic character make the studied BfQs very sensitive chromophores for sensors and optical switching devices.

## 1. Introduction

The nitrogen-containing fused heterocycles are the most abundant building block in medicinal chemistry, being used in the development of anticancer [1,2] and antifungal drugs [3,4,5]. The benzo-[f]-quinolines (BfQs) and their [3+2] cycloadducts stand as a versatile class of precursors for the pharmacological industry, in organic chemistry [1,2,3,4,5,6], and in obtaining dyes and fluorescent probes [7,8]. BfQs have two interesting structural features: a large delocalized electronic distribution, due to the polyheterocyclic structure, and a permanent dipole, due to the separated electron acceptor and donor moieties. Like all ylids, the 1,2-dipolar structure is achieved through the positive charge located on the nitrogen atom and the negative charge on the carbanion [9,10]. In addition, the electron withdrawing character and the hyperpolarizability of the quaternary amine endow BfQs with strong non-linear optical properties [11,12], which are now desired in photonics and information technologies.

The unusual photophysical properties of the charge-separated compounds such as BfQs stem from the low conjugation between the electron donor and acceptor units. The zwitterionic character is enforced in ground state and provokes pronounced negative solvatochromism. The molecular polarization is strongly dependent on the electric and coordinating properties of the solvent. In the solvation shell that surrounds the solute molecule, the solvent creates an internal electric field that influences the electronic states of the analyzed molecule [13,14]. The influence of the solvent on the electronic absorption spectra can be analyzed based on theoretical models [15,16,17] or by using empirical relations established between the wavenumber in the maximum of the absorption band and some solvent parameters [18,19,20,21,22,23]. The empirical dependences have the drawbacks of their limited applicability to similar chemical structures only.

**Scheme 1:**
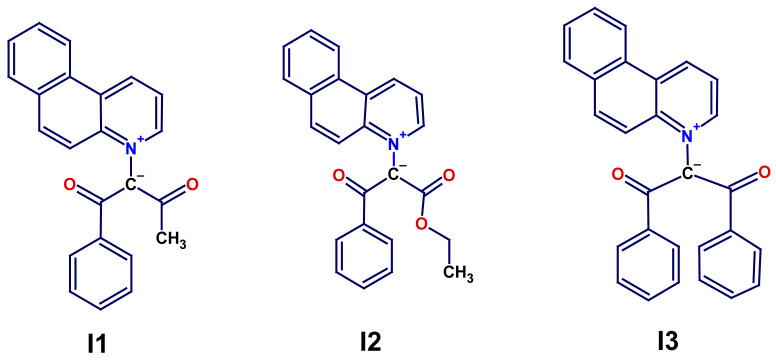
Chemical structure of the studied benzo-[f]-quinoline methylids.

In this work, we have addressed the negative solvatochromism of three BfQ derivatives by using a quasi-empirical procedure. The chemical structure of the benzo-f-quinolinium methylides allowed us to differentiate the solvatochromic effects according to differences in the short substituent. The compounds are as follows: benzo-[f]-quinolinium acetyl benzoyl methylid (**I1**), benzo-[f]-quinolinium carbethoxy benzoyl methylid (**I2**), and benzo-[f]-quinolinium dibenzoyl methylid (**I3**) [24,25] with structural features from Scheme 1. Some of the optical properties of **I1** were described in a previous study [25]. Because of their zwitterionic structure, **I1**–**I3** are able to distinguish between apolar and polar and/or protic solvents in terms of the hypsochromic or bathochromic behavior of their charge transfer absorption band.

In our study, the universal interactions are described by theoretically established functions of the refractive index and dielectric permittivity [26,27,28] and the specific interactions expressed by the empirical Kamlet-Abboud-Taft parameters [18,19,20,21,22,23]. Accordingly, the relation that shows the influence of the solvent on the electronic spectrum of the active molecule is as follows:

(1) $$\nu_{calc.}=\nu_{0}+C_{1}f\left(\varepsilon \right)+C_{2}f\left(n\right)+C_{3}\beta +C_{4}\alpha$$

where C_j_, j = 14, are the correlation coefficients; and f(ε)=(ε−1)/(ε+2) and $f\left(n\right)=\left(n^{2}-1\right)/\left(n^{2}+2\right)$ are the functions of dielectric permittivity and of the refractive index [26]. The second and third terms in Equation (1) were theoretically established. The *α* and *β* parameters describe the ability of the solvents to participate in specific interactions with the solute molecule by donating or accepting protons, respectively. The correlation coefficients depend on the electro-optical parameters of the solute. Equation (1) gives information about the ground state of the studied molecule, which generally is non-fluorescent. Details about the descriptors of the excited state of a solvated and non-fluorescent molecule can then be obtained by using various theories, like those of MacRae [17] or Abe [15,16]. We will apply the variational model [29,30,31], which is based on MacRae’s theory [17], in order to determine the electro-optical parameters in the excited state of **I1**, **I2** and **I3** ylids, according to the observed solvatochromism. In the second stage, we will use Abe’s theory of liquid [16,17] for determining the dipole moment and the polarizabilities in the excited state. The structure–optical property correlation was finally investigated by means of quantum chemical calculations with the DFT and TD-DFT methods.

## 2. Results and Discussion

### 2.1. Experimental Solvatochromism of ***I1***, ***I2*** and ***I3***

**Figure 1:**
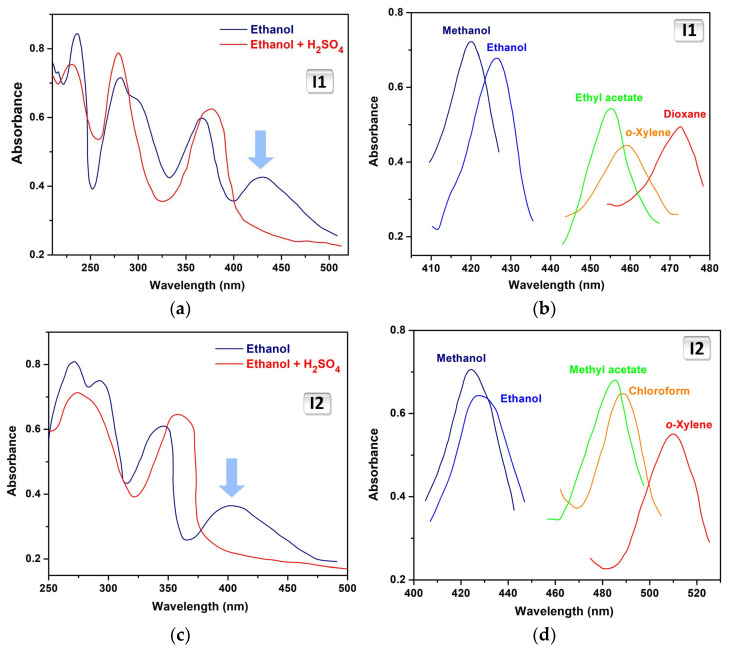

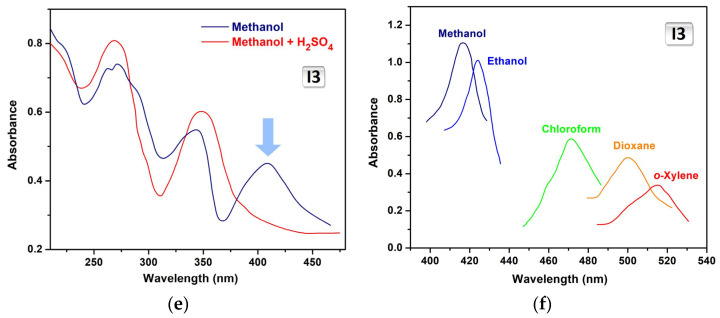
Exemplary electronic absorption spectra are as follows: (**a**) the UV-VIS region of **I1** in ethanol and acidified ethanol; (**b**) the ICT band of **I1** as a function of solvents of different polarity; (**c**) the UV-VIS region of **I2** in ethanol and acidified ethanol; (**d**) the ICT band of **I2** as a function of solvents of different polarity; (**e**) the UV-VIS region of **I3** in methanol and acidified methanol; (**f**) the ICT band of **I3** as a function of solvents of different polarity. The blue arrow indicates the main ICT band.

**Figure 2:**
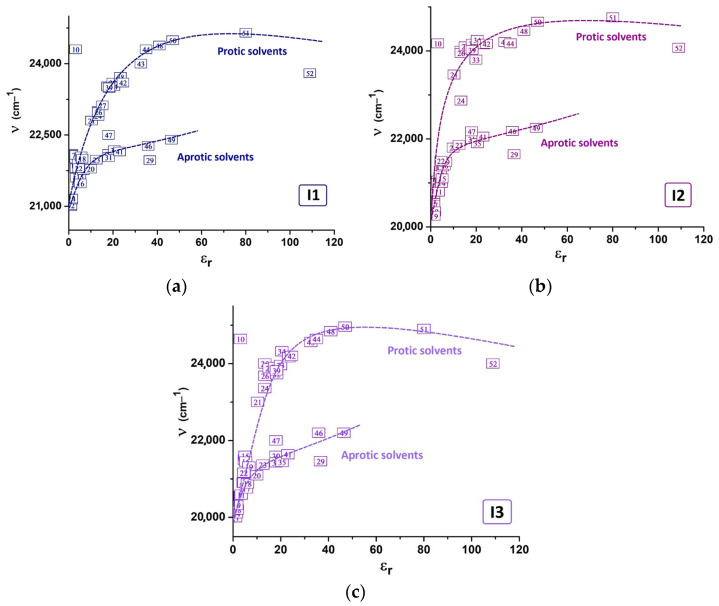
Plots of the maximum of the ICT band as a function of the dielectric permittivity of the solvent for (**a**) **I1**; (**b**) **I2**; (**c**) **I3**. The dotted lines are guides for the eye. The numbers on the experimental data points are the solvents’ numbering in Table 1.

The absorption spectra of **I1**, **I2** and **I3** have been recorded in a series of solvents with different characteristics in order to test their responses. As shown in Figure 1 for representative spectra in alcohols, **I1**, **I2** and **I3** have several electronic absorption bands. Two intense *π* → π* transitions are observed in the UV region around 270 nm and 350 nm, and a shallow one above 235 nm. Then n → π* transition in the Vis region is of a low intensity and is generated from an intramolecular charge transfer (ICT) [9,10,25,30,31,32], which makes it heavily dependent on the properties of the solvent, either electric or coordinative. The spectra of **I1**, **I2**, and **I3** in alcohol in comparison with those in acidified alcohol show the disappearance of the ICT band when protonation of the nitrogen atom blocks the electron transfer. The exemplary spectra in the visible region for low alcohols vs. solvents of low polarity in Figure 1b,d,f evidence the large blue shift in the ICT maximum. Its negative solvatochromism points to the stabilization of the molecule in apolar and aprotic solvents and a redistribution of the electronic cloud around the dipolar bond in polar solvents. The maximum of the ICT band for **I1**, **I2** and **I3** as a function of the solvent parameters for all solvents analyzed is listed in Table 1. As presented in Figure 2, the graphical representation of the ε value ν (cm^−1^) of the ICT maximum as a function of *f*(*ε*) for each BfQ splits the plot into two clear regions: the apolar/aprotic solvents, where the universal interactions dominate, and the polar/protic solvents, influenced by specific solvent–solute interactions.

In Figure 2, the experimental points corresponding to aprotic solvents are near the curve situated at smaller wavenumbers, and the representative points for protic solvents are situated near the curve situated at higher wavenumbers. The distance between the two curves estimates the energy of the quasi-chemical interactions in the protic solvents compared to the aprotic ones with the same macroscopic parameters. The separation of the points in Figure 2a–c is due to specific interactions of ylids with protic solvents shifting the ICT visible absorption band through the high wavelengths.

When both universal and specific interactions act in solutions, the Kamlet–Abboud–Taft solvent empirical parameters $\pi^{*}$, *α*, and *β* are used to express the shift in the electronic absorption band by multilinear equations of the following type:

(2) $$\nu_{calc.\;}=\nu_{0}+P\pi^{*}+A\alpha +B\;\beta \;$$

where $\nu_{0}$ is the value of the wavenumber in the maximum of the electronic band in a vacuum, *P* is the magnitude of the universal interactions, and *A* and *B* measure the strength of the specific interactions in which the solvent donates or receives protons from solute, respectively. The sign of the correlation coefficients shows the sense of the spectral shift.

Based on the data from Table 1, the results of the multiparametric regression are exhaustively listed in Table S1 and are briefly given through the following expressions:

***ν***(**I1**) = 21,079.48 + 922.02·π* + 1728.03·*α* + 622.60·β (R = 0.92) (3)

***ν***(**I2**) = 20,359.59 + 887.83·π* + 2260.64·*α* + 1677.93·β (R = 0.92), (4)

***ν***(**I3**) = 19,872.56 + 1220.10·π* + 2660.40·*α* + 1656.91·β (R = 0.95) (5)

Equations (3)–(5) show that in diluted solutions of the studied BFQs, the universal interactions are dominant in non-polar and non-protic solvents, while in protic solvents the specific interactions with receiving protons by the ylids become very important (Table 2).

The excited state dipole of **I1**, **I2** and **I3** was estimated based on both the results obtained by solvatochromic analysis of the spectral shifts in 48 solvents and the electro-optical parameters determined by quantum chemical calculations. The dependence of the *ν* value (cm^−1^) on the solvent parameters is given by Equation (1). By using the multilinear regression on Equation (1) and data from Table 1, the following dependencies were obtained:

(6) $$\nu_{calc.\;}\left(I1\right)=20,018+1145f\left(\varepsilon \right)+\;3978\;f\left(n\right)+226\;\beta +\;1888\;\alpha \;\;\left(R\;=\;0.919\right)$$

(7) $$\nu_{calc.\;}\left(I2\right)=19,982+1183\;f\left(\varepsilon \right)+1555\;f\left(n\right)+767\;\beta +2281\;\alpha \;\;\left(R\;=0.928\right)$$

(8) $$\nu_{calc.\;}\left(I3\right)=19,288+1524f\left(\varepsilon \right)+1983\;f\left(n\right)+907\;\beta +3032\;\alpha \;\left(R=0.970\right)$$

The variational method uses the coefficients *C*_1_ and *C*_2_, whose expressions contain the solvent descriptors, according to the following relations [33,34,35]:

(9) $$C_{1}r^{3}=2{µ}_{g}\left({µ}_{g}-{µ}_{e}cos\varphi \right)+3kT\left(\alpha_{g}-\alpha_{e}\right)$$

(10) $$(C_{1}+C_{2})\;r^{3}={µ}_{g}^{2}-{µ}_{e}^{2}+1.5\;\frac{I_{u}\;I_{v}}{I_{u}+I_{v}}\left(\alpha_{g}-\alpha_{e}\right)$$

The *C*_1_ and *C*_2_ coefficients are expressed in erg; *r* is the molecular radius (cm); *μ_g_* and *μ_e_* are the dipole moments in the ground and in excited state (Debye); *α_g_* and *α_e_* are the molecular polarizabilities (cm^3^); and *I_u_* and *I_v_* are the ionization potentials of the solute (*u*) and solvent (*v*) (in erg) (1 eV = 8066 cm^−1^; k = 1.38041 × 10^−16^ erg). The electro-optical parameters of **I1**, **I2** and **I3** calculated in vacuum (RCAM-B3LYP6-31G(d,p)/def2SV level) are listed in Table 3.

The variational model is based on the hypothesis of McRae [17,29,30,31] that the molecular polarizability could be considered constant in the process of light absorption. By using Equations (9) and (10) for C1 and C2, a relationship between the dipole moment in the excited state, *μ_e_*, and the angle between *μ_e_* and *μ_g_* (noted *φ*) can be determined. One obtains solutions of this relation as a function of the *φ* angle. By varying the angle and by calculating the polarizability for the obtained value of the dipole moment in the excited state, we can estimate that the electronic transition takes place when the polarizability in the excited state becomes equal to that in the ground state. By using the molecular descriptors from Table 3 and Equations (9) and (10), the variational model applied to **I1**, **I2** and **I3** resulted in the relations listed in Table 4 for the dipole moments in the excited state.

With the values obtained by the multilinear regression on Equation (1), the dipole moment and the polarizability of the spectrally active molecule (**I1**, **I2** and **I3**) in the excited state can be calculated. These values are close to the ground state, so in the limits of the measurement precision and of conditions in which the *C*_1_ and *C*_2_ parameters are determined, one can suppose that McRae’s hypothesis is accomplished in condition *φ* ≅0. Due to the chemical structure of the carbanion, we consider that the electronic transition upon light excitation occurs, in a first approximation, parallel to the ylid bond.

The variational model allows us to estimate numerically the values of *μ_e_*, if the angle *φ* between the dipoles in the ground and the excited state is varied and the dipole moment in the excited state is calculated for each value of *φ*. The method that considers the electronic transition in the absorption process taking place without any change in the molecular polarizability of the spectrally active molecule can be applied here only with some approximations. This is because the free term in the expression of the molecular polarizability vs. the squared excited dipole moment is smaller than that obtained by DFT calculations for the ground state.

Therefore, in the paradigm of the variational model and by using the relations from Table 4, we have determined the values of the dipole moments and polarizability of **I1**, **I2** and **I3** in the excited state for a number of values of the *φ* angle. The calculated values are given in Table 5, Table 6 and Table 7.

As it results from relation (5), the correlation coefficient *C*_1μ_ contains a term dependent only on the solute dipole moments and a term dependent on the solute polarizabilities in the two electronic states responsible for visible transition in absorption process. If one defines ratio R by relation (11), its variation with the angle *φ* can offer information about the visible absorption process. Equations (9) and (10) can be rewritten as follows:

(11) $$R=\frac{C_{1µ}}{C_{1\alpha }}=\frac{2{µ}_{g}\left({µ}_{g}-{µ}_{e}cos\varphi \right)}{1.5\;\frac{I_{u}\;I_{v}}{I_{u}+\;I_{v}}\left(\alpha_{g}-\alpha_{e}\right)}$$

**Figure 3:**
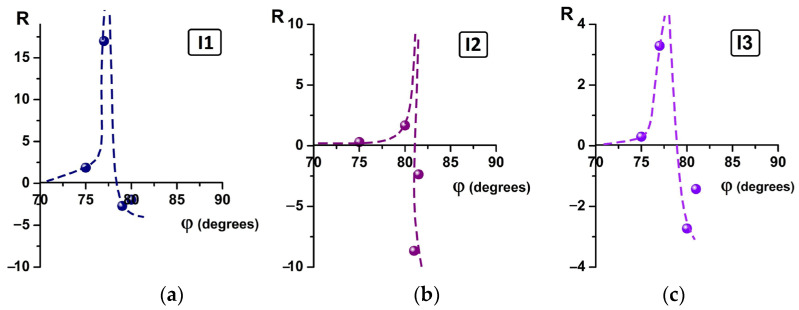
Graphical representation of the R values, calculated according to Equation (11), as a function of the angle between the dipole moments in the ground and in the excited state for (**a**) **I1**; (**b**) **I2**; (**c**) **I3**. The dotted lines are guides for the eye.

The *C*_1*μ*_ and *C*_1*α*_ and the *R* ratio can be calculated for each individual value of the *φ* angle and the corresponding values are listed in Tables S2–S4. The graphical representations of *R* vs. *φ* for **I1**, **I2**, and **I3** are presented in Figure 3.

The sign of R gives information about the electronic transitions. Figure 3 shows that R is a continuous function with a discontinuity near φ ≈ 80°. One can consider that the visible absorption can induce a charge redistribution determining such a high value of the angle between the dipole moments of solutes in the two electronic states participating in this process.

### 2.2. Abe’s Model of the Liquid

The results obtained above according to the variational model will be compared to those determined in the limits of the liquid theory developed by T. Abe [15,16]. In this model, the linear relationship between the values of the squared dipole moments in the ground and in the excited state is given by Equation (12):

(12) $${µ}_{e}^{2}\left(u\right)-{µ}_{g}^{2}\left(u\right)+\alpha_{e}\left(u\right)A=B$$

(13) $$A\;\left(\mathrm{erg}\right)=\frac{{µ}_{g}^{2}\left(v\right)+\frac{3}{2}\alpha_{g}\left(v\right)\frac{I_{g}\left(v\right)\left[I_{g}\left(u\right)-hc\nu_{s}\right]}{I_{g}\left(v\right)+I_{g}\left(u\right)-hc\nu_{s}}}{\frac{2}{3kT}{µ}_{g}^{2}\left(v\left(p\right)\right)+\alpha \left(v\right)}$$

(14) $$B\;\left(\mathrm{erg}\cdot {\mathrm{cm}}^{3}\right)=\frac{\frac{\nu_{0}-\nu_{s}}{C}+\left\{{µ}_{g}^{2}\left(v\right)+\frac{3}{2}\alpha_{g}\left(v\right)\frac{I_{g}\left(v\right)I_{g}\left(u\right)}{I_{g}\left(v\right)+I_{g}\left(u\right)}\right\}\;\alpha_{g}\left(u\right)}{\frac{2}{3kT}{µ}_{g}^{2}\left(v\left(p\right)\right)+\alpha \left(v\right)}$$

(15) $$C=\frac{16\pi^{3}N_{A}^{2}}{9}{\left(\frac{\rho_{v}}{M_{v}}\right)}^{\frac{2}{3}}\left\{{\left[{\left(\frac{M_{u}}{\rho_{u}}\right)}^{\frac{1}{3}}+{\left(\frac{M_{v}}{\rho_{v}}\right)}^{\frac{1}{3}}\right]}^{-4}+{\left[{\left(\frac{M_{u}}{\rho_{u}}\right)}^{\frac{1}{3}}+3{\left(\frac{M_{v}}{\rho_{v}}\right)}^{\frac{1}{3}}\right]}^{-4}+{\left[{\left(\frac{M_{u}}{\rho_{u}}\right)}^{\frac{1}{3}}+5{\left(\frac{M_{v}}{\rho_{v}}\right)}^{\frac{1}{3}}\right]}^{-4}+\ldots \right\}$$

**Figure 4:**
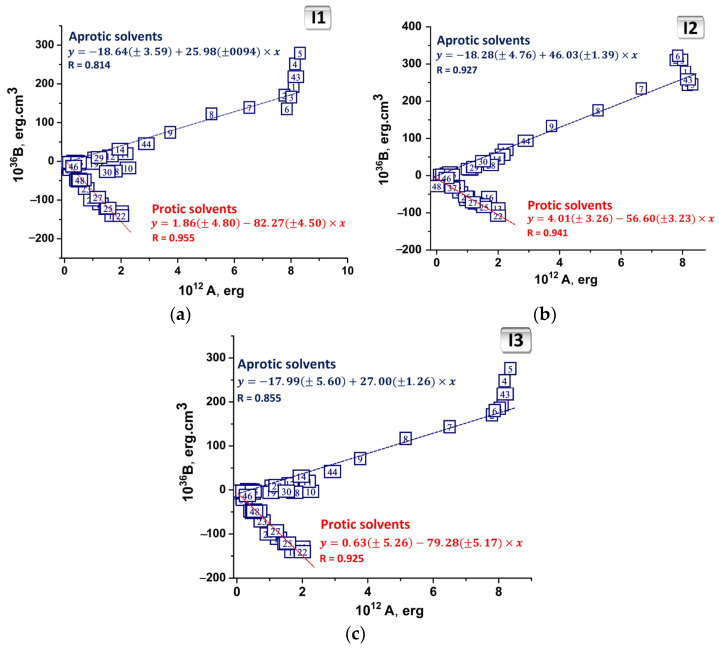
Graphical representation of the Abe parameters *B* vs. *A* for (**a**) **I1**; (**b**) **I2**, (**c**) **I3**. The experimental data are numbered according to the solvents’ numbering in Table 1. The dotted lines are the linear fit.

The *A*, *B*, and *C* parameters in relations (13) and (14) are functions of the molecular descriptors of the studied molecules, that is **I1**, **I2** and **I3**, and of the solvent parameters (Table S5). The other notations are designating the following: *M*—molar mass (g/mol); *ρ*—density (g/cm^3^); *T*—absolute temperature (K); $N_{A}$—Avogadro’s number; and *k*—gaseous constant. The values of *A*, *B*, and *C* for **I1**, **I2** and **I3** in the 48 solvents used in this work are listed in Tables S6 and S7. The graphical representation of *B* vs. *A* allows for the determination of the polarizability of the excited state from the slope, while the intercept represents the difference ${µ}_{e}^{2}\left(u\right)-{µ}_{g}^{2}\left(u\right)$. Accordingly, Figure 4 shows the *B* vs. *A* plot for **I1**, **I2**, and **I3**.

In Figure 4, two different curves are seen for each of the analyzed BfQs: one with a positive slope that follows the regular trend for most of ICT compounds, and the other with a steep negative slope, which is highly uncommon. The linear increase in B occurs in apolar and non-H-bonding solvents, from which the dipole moment in the excited state (Equation (12)) is determined as being lower than in the ground state (Table 8). In polar and coordinating solvents, the calculated values of the dipole moment in the excited state are higher than in the ground state.

The calculated values for the excited state dipole moments and polarizabilities of the studied BfQ molecules were obtained based on the computed corresponding parameters for the ground state of the isolated molecules in a vacuum. For this reason, the obtained values could be affected by solvent influence on the molecular parameters of the ground state. The specific interactions between ylids and the protic solvents, for example, increase the ground state dipole moments of ylids. The data obtained based on the Abe model are affected by the hypotheses in which this model is developed. So, one considers that the ground and excited dipole moments are collinear and also that the specific solute–solvent interactions are neglected. Nevertheless, the decrease in the dipole moment by excitation in the absorption process, common to the studied ylids, is evidenced both in the data computed based on the variational model and also in the Abe model.

### 2.3. Quantum Chemical Investigations of ***I1***, ***I2*** and ***I3***

**Figure 5:**
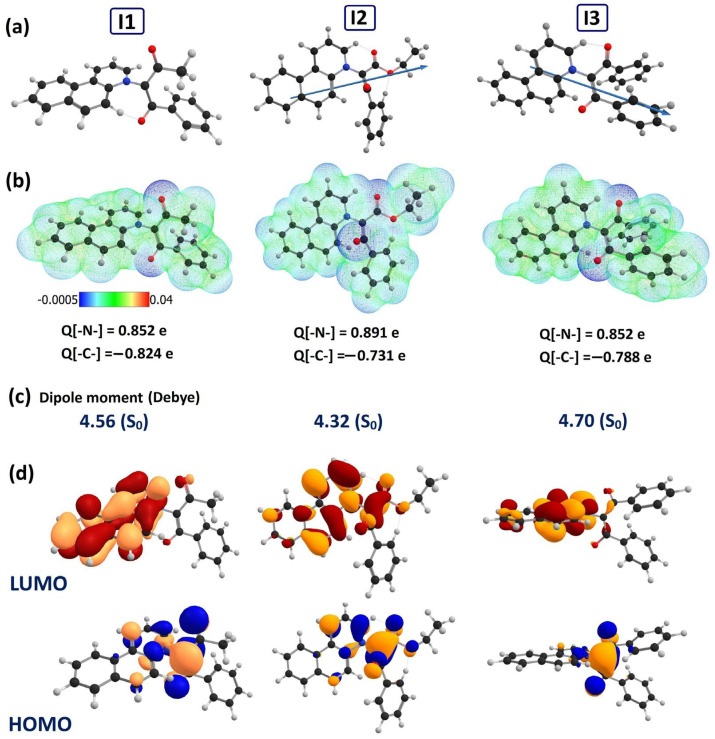
(**a**) Optimized molecular structures of **I1**, **I2**, and **I3** in ground state in vacuum. (**b**) Electrostatic potential surfaces of **I1**, **I2**, and **I3**; Q represents Mulliken charges for acceptor [-N] and donor [-C-] sites. (**c**) Values of dipole moments in ground state. (**d**) Frontier molecular orbitals HOMO and LUMO. Calculations: RCAM-B3LYP6-31G(d,p)/def2SV. Color code: positive lobes - orange; negative lobes - blue (HOMO) and red (LUMO).

The structural features of the studied benzo-[f]-quinoline methylids were investigated at the RCAM-B3LYP6-31G(d,p)/def2SV level of theory. The geometry in the relaxed ground state in vacuo for **I1**, **I2**, and **I3** is shown in Figure 5. Irrespective of the substituent on the ylidic carbon, the molecules are generally non-planar, with the two arms twisted with respect to each other and with the plane of the fused heterocycles. The twisting degree is variable and the ground energy does not differ too much from one rotamer to another. The charge distribution on the donor moiety is slightly different according to the kind of short substituent on the ylidic carbon. From carbethoxy to benzoate as substitution groups, the Mulliken charge on the carbanion decreased from **I1** (−0.824 e) to **I2** (−0.734 e) and increased again in **I3** (−0.788 e). The magnitude of the dipole moment follows the same trend, but overall, the dipole moment in the ground state is around 4.5 D on average. The electrostatic potential (Figure 5b) does not differ from one BfQ to another, having the nucleophilic activity concentrated on the carbanion oxygen atoms. In the ground state, the charge is accumulated on the carbanion, as HOMO orbitals show (Figure 5d), and becomes displaced on the fused heterocycles in LUMO. The intramolecular charge transfer in **I1**, **I2** and **I3** occurs from HOMO to LUMO, which is in agreement with previous studies on cycloimmonium ylids [29,30,31,32,33,34].

Insights into the electronic absorption properties of **I1**, **I2** and **I3** were further obtained by TD-DFT methods with the same functional and basis set. The calculations were made for a vacuum and two representative solvents, cyclohexane (CHX) and water. The TD-DFT analysis revealed that the spectra profile and especially the intensity of the ICT band are completely dependent on the conformation of the molecule. The free rotation around the single bond between the heterocycle and ylid carbon, and the many degrees of freedom at the two substituents, lead to a bunch of conformations that affect the energetics of the molecule to a certain degree. The most mobile group is the short substituent, and its orientation relative to the heterocycle is the decisive factor in the absorption process. The orientation of the benzenoid substituent is always orthogonal on the heterocycle, due to steric hindrance, so it does not greatly affect the intensity of the ICT process, but rather the vibronic structure of the intense *π* → π* band around 250 nm.

**Figure 6:**
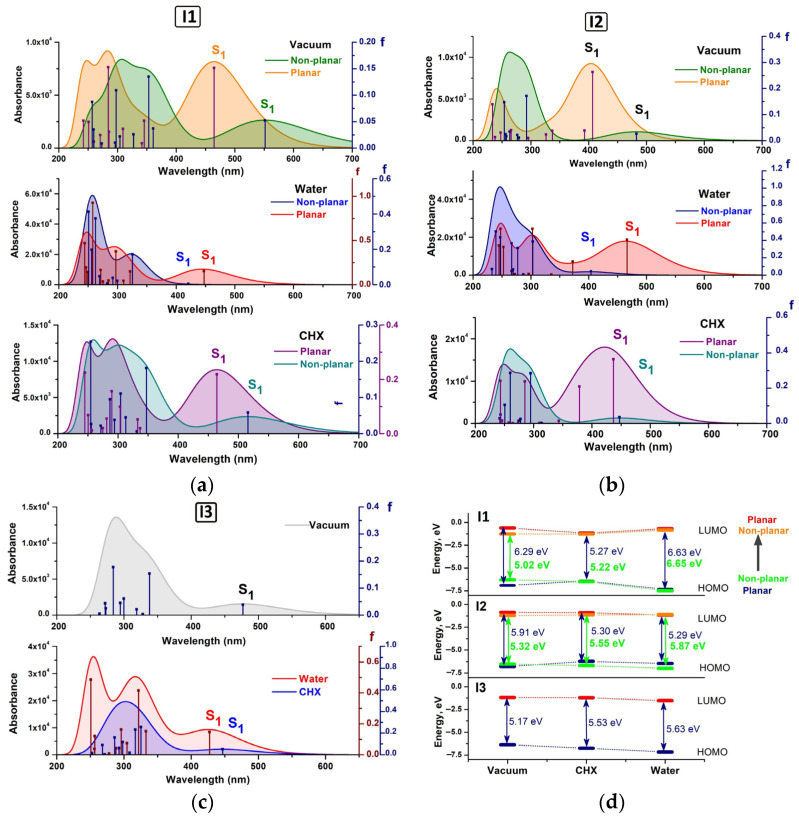
TD-DFT analysis of the electronic transitions in a vacuum as compared to the implicit solvents cyclohexane and water, and the oscillator strength (f) for (**a**) **I1**; (**b**) **I2**; (**c**) **I3**. (**d**) Energy levels of the frontier molecular orbitals of **I1**, **I2** and **I3** in the three considered media.

The influence of the molecular orientations is strong for **I1** and **I2**. Table 9 lists the values of the dipole moments and Mulliken charges in the ground state for the planar and twisted conformations for **I1**–**I3**. From linear to twisted, the dipole moment in the ground state increases and the electric charge on the N atom decreases. Also, from CHX to water, the dipole moment becomes higher and the Mulliken charge on the nitrogen atom becomes smaller. In very close connection with these parameters, the profiles of the calculated UV-Vis spectra differ more in terms of their molecular conformation and less in regard to the type of the solvent. Figure 6a,c illustrates the UV-Vis spectra of **I1**, **I2** and **I3** for the planar and the orthogonal orientation of the short substituent at the ylide carbon atom relative to the plane of the polyheterocycle, and the oscillator strength of the electronic transitions, f. Overall, the calculated profile is similar to the experimental spectra, although the band around 344–366 nm of all BFQs was not captured by DFT calculations, whatever the method used. The ICT wavelength maxima were slightly overestimated in water in comparison with the experimental values, and were underestimated in cyclohexane. Yet, the ICT is at its maximum when the short substituent at the ylid carbon is oriented almost in plane with the fused heterocycle. When the substituent is twisted out of the plane of the molecule, the ICT band is weak and the maximum is bathochromically shifted towards 500 nm and beyond for vacuum and non-polar solvents, and hypsochromically shifted in protic solvents. For **I3**, the two substituents pose a strong hindrance with respect to the heterocycle and they are generally parallel to each other and perpendicular to the plane of the heterocycle. Therefore, there are no great differences in molecular conformations nor in the absorption properties between them. Figure 6c presents the representative UV-VIS spectra of **I3** as an average for the electronic absorption. One can state that the orientation of substituents can be somewhat predicted by analyzing the profile of the UV-Vis spectrum, the intensity, and the position of the ICT band. We conclude that the experimental spectra, where the ICT band is of low intensity, consists of overlapping contributions from molecules with twisted conformations at the polar bond and with many rotamers within the short substituent.

The lowest excited state, S_1_, is basically described by a HOMO–LUMO transition of low oscillator strength in non-polar cyclohexane, and by extension, in a vacuum, and of high oscillator strength in polar solvents. In water, the calculated electronic spectra are higher in intensity but with a weaker ICT band, which suggests a redistribution of the electronic cloud by interaction with the water dipoles. In conjunction with the hole–electron distribution analysis presented in Table 10 and Table 11, the HOMO–LUMO transition arises from a charge transfer from the carbanion to the fused heterocycle, a transition that occurs in whatever medium is analyzed. One can see that the carbonyl groups also do participate in transition, as the hole has a nodal plane along the oxygen atoms. The electron distribution is localized mainly near the nitrogen, where most of the charge is transferred after excitation. We observe that the transferred charge is distributed on half of the heterocycle. Accordingly, the charge difference upon electronic transition follows the profile of the hole–electron localization, where the electronic density of the ylid carbon decreases and further increases in the heterocycle.

The comparison of the calculated energy of the frontier molecular orbitals between cyclohexane and water, shown in Figure 6d, confirms the negative solvatochromism by lowering the HOMO energy for any of BfQs and enlarging the HOMO–LUMO gap. There is an expected difference in the energy of HOMO and LUMO between the planar and the twisted conformations. The HOMO and LUMO levels are lower for non-planar molecules and with a higher energy gap than those of planar ones.

The TD-DFT analysis of the S_1_/ICT state, presented in Table 10 for **I1** as a representative example, shows that the electronic charge is reversed as compared to the ground S_0_ state, as the nitrogen atom carries a negative charge after electronic excitation. The same situation appears for **I2** and **I3** and is not presented here. The charge transfer occurs irrespective of the conformation, but is strongly favored when the carbonyl group in the substituent arm aligns with the aromatic structure in the heterocycle. It leads to a higher negative charge on nitrogen in S_1_/ICT than in the twisted molecule, according to the values of Q [-N-] in Table 10. But the values of the dipole moments listed in Table 10 for **I1** indicate that the excited planar molecule is less polar than the excited twisted molecule. The dipole moment in S_1_/ICT is twice the magnitude in water as compared to cyclohexane.

Table 11 contains the analysis of the electronic excitation for **I2** and **I3** in planar conformation in order to focus on the intense ICT process. As was explained above, the charge transfer for planar and twisted **I2** is similar to that in **I1**. For both **I2** and **I3**, there is a net separation in the centers for electronic charge between donor and acceptor units. As in **I1**, the transferred electronic charge is restricted to half of the heterocycle, which is near the nitrogen.

In contrast with other cycloimmonnium ylids, whose hole and electron distribution have changed according to the type of solvent [33,34], the hole of the BfQ remains mainly located on the carbanion and the electrons transfer to nitrogen and the nearest aromatics. The ICT character of the S_0_ → S_1_ transition is confirmed for any solvent and does not seem to be affected, even in strongly coordinating solvents like water. Summing up the above observations, the negative solvatochromism of zwitterionic benzo-[f]-quinolinium methylids is given by three key factors: (i) alkyl vs. aryl units as terminal groups in the short substituent, because the less voluminous and virtually linear alkyl chain will allow the free rotation around the polar bond without the steric hindrance imposed by bulk aromatic rings; (ii) the conformation around the polar bond, which is mandatory for the ylids with the acetyl and the carbethoxy substituents, and the electron transfer is maximal where the carbonyl group in these substituents is in the vicinity of the polyheterocycle; and iii) the polarity and coordinating properties of the solvent.

## 3. Materials and Methods

The synthesis, structural characterization, and analysis of the electro-optical properties of **I1**, **I2**, and **I3** followed the established procedure [24]. Elemental analysis was performed on an Elemental Exeter Analytical CE 440 apparatus (Exeter Analytical Inc., Coventry, UK). The ^1^H-NMR and ^13^C-NMR measurements were made on a high-resolution liquid NMR 400 MHz spectrometer Bruker Neo-1 (Bruker BioSpin GmbH, Rheinstetten, Germany) for a direct detection probe, with 5 mm Quadra nuclei probes, or QNPs (four nuclei, 1H/13C/19F/29Si). The FTIR spectra were recorded as KBr pellets with a Bruker Vertex 70 spectrometer (Bruker Optics GmbH, Ettlingen, Germany) between 4000 and 400 cm^−1^ at a 2 cm^−1^ resolution.

Benzo-[f]-quinolinium-acetyl-benzoyl methylid (**I1**). Orange powder. Anal. calcd for: C_23_H_17_NO_2_: 81.42% C; 5.01% H; 4.13% N. Found: 81.83% C; 5.39% H; 4.61% N. M. p. 136 °C. ^1^H-NMR (DMSO-d_6_, 400 MHz), *δ* (ppm): 2.89 (s, 3H, CH_3_); 7.22–7.24 (d, 2H, CHAr); 7.39–7.40 (d, 1H, CHAr); 7.45–7.47 (d, 2H, CHAr); 7.57–7.59 (d, 2H, CHAr); 7.65–7.67 (d, 1H, CHAr); 7.88–7.91 (d, 2H, CHAr); 8.12–8.14 (d, 1H, CHAr); 8.37–8.40 (d, 1H, CHAr); 8.83–8.85 (d, 1H, CHAr); 8.99–9.02 (d, 1H, CHAr). ^13^C-NMR (DMSO-d_6_, 400 MHz), *δ* (ppm): 25.86 (CH_3_); 125.3; 126.7; 127.2; 127.5; 127.9; 128.2; 128.5; 128.9; 129.1; 130.1; 130.6; 130.9; 131.1; 134.4; 134.7; 134.9; 135.4; 140.8; 142.6 (Ar); 139.1 (C^-^); 181.6; 193.4 (C=O). FTIR (KBr) *ν*_max_, cm^−1^: 3015w (*ν*CH_arom_), 2919w (*ν*CH), 2838w (*ν*CH), 1720s (*ν*C=O), 1703m (*ν*C=O), 1620m (*ν*C=C), 1595s (*ν*C=C), 1545s (*ν*C=C), 1505s (*ν*C=C), 1460m (*δ*CH), 1404m, 1396m (*δ*CH_3_), 1232m, 862m, 803m (*δ*CC_arom_), 730m (*δCC*/(*δCH*).

Benzo-[f]-quinolinium-carbethoxy-benzoyl methylid (**I2**). Orange powder. Anal. calcd. for: C_24_H_19_NO_3_: 78.05% C; 5.15% H; 3.79% N. Found: 78.44% C; 5.50% H; 4.26% N. M. p. 142–143 °C. ^1^H-NMR (DMSO-d_6_, 400 MHz), *δ* (ppm): 1.35–1.37 (m, 3H, CH_3_); 4.52–4.54 (m, 2H, CH_2_); 7.19–7.21 (d, 2H, CHAr); 7.31–7.33 (d, 1H, CHAr); 7.43–7.46 (d, 2H, CHAr); 7.67–7.69 (d, 2H, CHAr); 7.75–7.77 (d, 1H, CHAr); 7.84–7.85 (d, 2H, CHAr); 8.17–8.19 (d, 1H, CHAr); 8.42–8.44 (d, 1H, CHAr); 8.86–8.89 (d, 1H, CHAr); 9.00–9.03 (d, 1H, CHAr). ^13^C-NMR (DMSO-d_6_, 400 MHz), *δ* (ppm): 26.11 (CH_3_); 61.55 (CH_2_); 125.8; 127.1; 127.5; 127.7; 128.1; 128.6; 128.9; 129.2; 129.4; 130.3; 130.9; 131.6; 131.9; 134.6; 135.2; 135.7; 135.8; 141.3; 142.9 (Ar); 184.3; 195.1 (C=O); 139.9 (C^−^). FTIR (KBr) *ν*_max_, cm^−1^: 3025w (*ν*CH_arom_), 2998w (*ν*CH_arom_), 2931w (*ν*CH), 2858w (*ν*CH), 1725s (*ν*C=O), 1705m (νC=O), 1615m (*ν*C=C), 1603m (*ν*C=C), 1598s (*ν*C=C), 1560s (*ν*C=C), 1505s (*ν*C=C), 1453m (*δ*CH), 1420s (*ν*C=C), 1383m, 1378s (*δ*CH_3_), 1327m, 797m (*δ*CC), 737m (*δCC*/(*δCH*).

Benzo-[f]-quinolinium-dibenzoyl methylid (**I3**). Orange crystals (recrystallization from methanol). Anal. calcd for: C_28_H_19_NO_2_: 83.79% C; 4.74% H; 3.49% N. Found: 84.15% C; 5.11% H; 3.86% N. M. p. 230–231 °C. ^1^H-NMR (DMSO-d_6_, 400 MHz), *δ* (ppm): 7.25–7.27 (d, 2H, CHAr); 7.34–7.37 (d, 1H, CHAr); 7.42–7.44 (d, 4H, CHAr); 7.51–7.53 (d, 4H, CHAr); 7.60–7.62 (d, 2H, CHAr); 7.83–7.85 (d, 2H, CHAr); 8.22–8.24 (d, 1H, CHAr); 8.46–8.48 (d, 1H, CHAr); 8.79–8.81 (d, 1H, CHAr); 9.11–9.13 (d, 1H, CHAr). ^13^C-NMR (DMSO-d_6_, 400 MHz), *δ* (ppm): 126.6; 126.9; 127.4; 127.6; 127.8; 128.3; 128.5; 128.7; 128.8; 128.9; 129.9; 130.3; 130.5; 130.8; 131.3; 131.6; 131.7; 131.9; 134.6; 134.8; 135.6; 135.9; 136.2; 142.8; 143.3 (Ar); 194.7; 194.9 (C=O); 138.8 (C^−^). FTIR (KBr) *ν*_max_, cm^−1^: 3049w (*ν*CH_arom_), 3021w (*ν*CH_arom_), 2932w (*ν*CH), 2854w (*ν*CH), 1748s (*ν*C=O), 1735m (*ν*C=O), 1627m (*ν*C=C), 1603m (*ν*C=C), 1598s (*ν*C=C), 1556s (*ν*C=C), 1498s (*ν*C=C), 1462m (*δ*CH), 1327m (*δ*C=C), 874m (*δ*CH_arom_), 797m (*δ*CC_arom_), 735m (*δCC*/(*δCH*), 650m.

The UV-Vis absorption spectra were recorded with an Analytic Jena Specord Plus-5 spectrophotometer (Jena GmbH, Germany), in suprasil quartz cuvettes of a 10 mm pathlength. The solvents used were of spectral grade and from Merck (Merck KGaA, Darmstadt, Germany), Sigma Aldrich (Saint-Louis, MO, USA) and Chemopar (Iasi, Romania). The solvent parameters, which are microscopic (α and β) and macroscopic (ε and n), were obtained from previous studies [10,25,29,30,31,32,33,34] and from the literature [17,18,19,20,21,22,23].

The electronic properties of the studied BfQs were investigated in the DFT and TD-DFT methods with the Gaussian 09W version A.01 software [35]. The relaxed geometries were calculated in vacuo and in the presence of an implicit solvent method without symmetry constraints, firstly with the PM3MM method, and refined with the long-range corrected functional RCAM-B3LYP with the 6-31G(d,p) basis set and def2SVfitting model. The effects of electron correlation and long-range correction on the optical properties were taken into account [36]. The calculations were made for cyclohexane and water as the solvents of choice. The minimum on the potential energy surface in every case was verified by calculating the harmonical vibrational frequencies. The electronic excitation was analyzed by using the Multifwn 3.8 software [37].

## 4. Conclusions

The values of the dipole moments of the studied BfQs in the S_1_/ICT state, estimated by two methods, the variational model and the Abe model, are smaller in comparison to those calculated for the ground state, proving the decrease in the solvation energy in the ICT process due to light absorption. The differences between the obtained values of the excited state dipole moments in the two models are due to the hypotheses in which these methods were developed. The studied molecule BfQs interact with the protic solvents by means of hydrogen bonds, which increase the moment dipole in the ground state and change the energetics of the excited state. The small twist angle favored the ICT transition. The structure of the short substituent influenced the energy levels of the molecules, as the carbethoxy substituent raised the HOMO level in comparison to the shorter acetyl chain and compressed the energy gap. The HOMO–LUMO gap progressively increased from non-polar to polar solvents. The clear negative solvatochromism and the electro-optical properties lead to interesting applications of **I1**, **I2** AND **I3** as a sensor for harmful reagents and gases. Further studies of non-linear optical characteristics may recommend BfQs as useful in optical switching technologies.

## Figures and Tables

**Table 1:** Solvent parameters and the position of the ICT band of **I1**, **I2** and **I3**.

No.	ε	*f(ε)*	*f(n)*	π*	*β*	*α*	ν (nm) I1	ν (nm) I2	ν (nm) I3
1	n-Heptane	0.227	0.24	−0.08	0	0	476	477	500
2	n-Hexane	0.241	0.229	−0.04	0	0	476	477	496
3	Dioxane	0.286	0.251	0.55	0.37	0	473	475	486
4	Carbon tetrachloride	0.292	0.274	0.28	0.1	0	472	476	497
5	Benzene	0.299	0.295	0.59	0.1	0	454	485	488
6	*p*-Xylene	0.299	0.291		0.13	0	452	487	490
7	1,3,5-Trimethylbenzene	0.302	0.293	0.41	0.13	0	453	488	487
8	*o*-Xylene	0.302	0.292	0.41	0.11	0	459	491	495
9	Toluene	0.348	0.297	0.54	0.11	0	459	494	492
10	Propanoic acid	0.434	0.239		0.45	1.12	411	414	406
11	Trichloroethylene	0.448	0.282	0.48	0.05	0	463	481	485
12	Methoxybenzene	0.524	0.302	0.73	0.32	0	456	472	478
13	1,2-Dibromoethane	0.538	0.313		0	0	458	476	478
14	Chloroform	0.552	0.267	0.69	0.1	0.2	465	476	465
15	Butyl acetate	0.577	0.24	0.46	0.45	0	462	474	463
16	3-Methylbutyl acetate	0.589	0.241	0.71	0.07	0	465	468	466
17	Chlorobenzene	0.605	0.307	0.71	0.07	0	453	467	482
18	Ethyl acetate	0.625	0.228	0.55	0.45	0	454	467	479
19	Methyl acetate	0.655	0.221	0.6	0.42	0	459	465	469
20	Dichloromethane	0.727	0.256	0.82	0.1	0.2	459	458	474
21	Octan-1-ol	0.745	0.258	0.4	0.81	0.77	438	426	435
22	1,2-Dichloroethane	0.752	0.264	0.81	0.1	0	459	465	472
23	Pyridine	0.79	0.299	0.87	0.64	0	455	457	468
24	Hexan-1-ol	0.839	0.252	0.04	0.84	0.8	436	437	428
25	Phenylmethanol	0.804	0.311	0.98	0.52	0.6	435	416	416
26	Cyclohexanol	0.824	0.276	0.45	0.84	0.66	435	417	422
27	Pentan-1-ol	0.826	0.241	0.4	0.86	0.84	432	415	418
28	Butan-1-ol	0.833	0.242	0.47	0.84	0.84	425	416	418
29	1-Phenylethanone	0.833	0.312	0.9	0.49	0.04	455	462	466
30	Butan-2-one	0.85	0.232		0.48	0.06	452	454	462
31	Cyclohexanone	0.85	0.269	0.76	0.53	0	454	454	466
32	2-Methylpropan-1-ol	0.852	0.239	0.4	0.84	0.69	426	414	422
33	Propan-2-ol	0.852	0.234	0.48	0.84	0.76	423	420	417
34	Propan-1-ol	0.866	0.239	0.52	0.9	0.84	425	412	411.
35	Acetone	0.868	0.222	0.62	0.48	0.08	450	456	466
36	Propane-1,2-diol	0.882	0.259		0.8	0.58	421	414	414
37	4-Hydroxy-4-methylpentan-2-one	0.883	0.253	0.65	0.55	0	425	416	420
38	Pentane-2,4-dione	0.892	0.269		0.6	0	451	453	462
39	Ethanol	0.895	0.221	0.86	0.75	0.86	424	414	413
40	Methanol	0.909	0.203	0.6	0.66	0.98	416	413	407
41	Propane-1,3-diol	0.915	0.263		0.85	0.77	411	414	406
42	Acetonitrile	0.921	0.219	0.75	0.4	0.19	449	451	450
43	Dimethylformamide	0.924	0.258	0.88	0.69	0	444	451	454
44	Ethane-1,2-diol	0.93	0.259	0.9	0.52	0.9	410	409	402
45	Dimethylsulfoxide	0.946	0.281	1	0.76	0	446	449	450
46	Propane-1,2,3-triol	0.948	0.28		1.14	0.7	408	405	400
47	Water	0.964	0.206	1.09	0.47	1.17	405	404	402
48	Formamide	0.973	0.271	0.97	0.48	0.71	420	415	416

**Table 2:** Percentage contributions of the solvatochromic parameters in the Kamlet–Taft approach.

Compound	P_π*_ (%)	P_α_ (%)	P_β_ (%)
**I1**	28.17	52.80	19.02
**I2**	18.39	46.83	34.76
**I3**	22.03	48.04	29.92

**Table 3:** The electro-optical parameters of isolated **I1**, **I2**, and **I3** molecules, determined by the DFT method.

Parameter	I1	I2	I3
$E_{\mathrm{HOMO}}$ (eV)	−5.21	−5.21	−5.23
$E_{\mathrm{LUMO}}$ (eV)	−2.36	−2.53	−2.39
${µ}_{g}\;\left(D\right)$	4.56	4.319	4.708
$\alpha_{g\;}\left({\overset{\cdot }{A}}^{3}\right)$	41.077	45.12	34.50
A (Å^2^)	306.67	328.43	360.88
V (Å^3^)	313.87	339.65	368.71
r^3^ (Å^3^)	28.94	29.86	28.79

**Table 4:** Equations for determining the dipole moment in the excited state of **I1**, **I2** and **I3** molecules by the variational model.

**I1**	$C_{1\;}r^{3}=6.5830$	$0.01506{µ}_{e}^{2}-9.12{µ}_{e}cos\varphi +35.1347=0$
	$(C_{1}+C_{2})\;r^{3}=29.4539$	
	$\alpha_{e}=40.0125-0.1229{µ}_{e}^{2}$	${µ}_{1,2}^{e}=\frac{9.12cos\varphi \pm \sqrt[{2}]{9.12cos\varphi -2.1165}}{0.03012}$
**I2**	$C_{1\;}r^{3}=11.1686$	$0.01506{µ}_{e}^{2}-8.64{µ}_{e}cos\varphi +26.3688=0$
	$(C_{1}+C_{2})\;r^{3}=20.3918$	
	$\alpha_{e}=44.9074-0.1229{µ}_{e}^{2}$	${µ}_{1,2}^{e}=\frac{8.64cos\varphi \pm \sqrt[{2}]{{\left(8.64cos\varphi \right)}^{2}-1.5885}}{0.03012}$
**I3**	$C_{1\;}r^{3}=8.7163$	$0.015{µ}_{e}^{2}-9.416{µ}_{e}cos\varphi +35.6459=0$
	$(C_{1}+C_{2})\;r^{3}=20.0579$	
	$\alpha_{e}=34.766-0.1225{µ}_{e}^{2}$	${µ}_{1,2}^{e}=\frac{8.7163cos\varphi \pm \sqrt[{2}]{{\left(9.416cos\varphi \right)}^{2}-2.1388}}{0.03014}$

**Table 5:** The dipole moments and polarizabilities of **I1** in the first excited state, calculated in the limits of the variational model.

*φ* (degree)	${µ}_{e}\;\left(D\right)$	$\alpha_{e}\;(A^{3}$)	*φ* (degree)	${µ}_{e}\;\left(D\right)$	$\alpha_{e}\;(A^{3}$)
0	3.88	38.16	76	18.18	−0.61
20	4.13	37.92	77	20.09	−9.59
40	5.08	36.84	78	22.58	−22.65
60	7.91	32.32	79	26.07	−43.54
75	16.65	6.01	80	31.81	−84.31

**Table 6:** The dipole moments and polarizabilities of **I2** in the first excited state, calculated in the limits of the variational model.

*φ* (degree)	${µ}_{e\;}\left(D\right)$	$\alpha_{e}\;(A^{3}$)	*φ* (degree)	${µ}_{e}\;\left(D\right)$	$\alpha_{e}\;(A^{3}$)
0	3.07	43.75	78.8	18.93	0.87
20	3.27	43.60	78.9	19.19	−0.33
40	3.94	43.00	79	19.45	−1.60
60	6.24	40.12	80	21.99	−14.54
75	12.92	24.41	81.	28.67	−56.09
78.5	1.72	18.21	81.5	35.46	−109.5

**Table 7:** The dipole moments and polarizabilities of **I3** in the first excited state, calculated in the limits of the variational model.

*φ* (degree)	${µ}_{e}\;\left(D\right)$	$\alpha_{e}\;(A^{3}$)	*φ* (degree)	${µ}_{e}\;\left(D\right)$	$\alpha_{e}\;(A^{3}$)
0	3.81	32.99	77		
20	4.06	32.75	78	21.87	−23.85
40	4.99	31.71	79	25.10	−42.42
60	7.76	27.38	80	30.13	−76.44
75	16.25	2.41	81	43.24	−194.29

**Table 8:** The calculated values of the dipole moment and polarizabilities in the excited state of **I1**, **I2** and **I3**, according to Abe’s model of the liquid.

BfQ	Solvent	Equation	${µ}_{e}\;\left(D\right)$	$\alpha_{e}\;\left(cm^{-1}\right)$
**I1**	Aprotic	B = (−18.64 ± 3.59)+ (25.98 ± 0.94)*A*	1.47	25.98
	Protic	B = (1.86 ± 4.80) − (82.27 ± 4.50)*A*	4.76	−82.27
**I2**	Aprotic	B = (−23.28 ± 4.76) + (46.03 ± 1.39)*A*	4.02	46.03
	Protic	B = (4.01 ± 3.26) − (56.60 ± 3.23)*A*	6.59	−56.60
**I3**	Aprotic	B = (−17.99 ± 5.60) + (27.00 ± 1.26)*A*	4.92	27.00
	Protic	B = (0.63 ± 5.26) − (79.28 ± 5.17)*A*	4.77	−79.28

**Table 9:** Calculated dipole moments and Mulliken charges on the donor and acceptor atoms for the ground state of **I1**, **I2** and **I3** in vacuum, cyclohexane, and water for the two extreme rotamers.

BfQ	Solvent	Dipole Moment, (D)	Dipole Moment, (D)	Mulliken Charges (e)	Mulliken Charges (e)
		Planar	Non-Planar	Planar	Non-Planar
**I1**	Gas	4.256	4.742	Q[-N-] = 0.852 Q[-C-] = −0.824	Q[-N-] = 0.861 Q[-C-] = −0.769
	CHX	4.470	4.76	Q[-N-] = 0.898 Q[-C-] = −0.781	Q[-N-] = 0.833 Q[-C-] = −0.809
	Water	6.055	5.968	Q[-N-] = 0.880 Q[-C-] = −0.800	Q[-N-] = 0.793 Q[-C-] = −0.770
**I2**	Vacuum	4.209	5.050	Q[-N-] = 0.901 Q[-C-] = −0.731	Q[-N-] = 0.865 Q[-C-] = −0.786
	CHx	4.32	6.094	Q[-N-] = 0.892 Q[-C-] = −0.718	Q[-N-] = 0.837 Q[-C-] = −0.789
	Water	6.283	7.189	Q[-N-] = 0.865 Q[-C-] = −0.762	Q[-N-] = 0.796 Q[-C-] = −0.776
**I3**	Vacuum	4.526		Q[-N-] = 0.852 Q[-C-] = −0.788	
	CHx	4.825		Q[-N-] = 0.842 Q[-C-] = −0.812	
	Water	6.494		Q[-N-] = 0.821 Q[-C-] = −0.775	

**Table 10:** Characteristics of **I1** in the S_1_/ICT state in two different conformations, the dipole moment, the hole (blue) – electron (green) distribution during excitation, and the charge density difference between states for the S_0_ → S_1_/ICT transition (blue and green parts correspond to a decrease and an increase, respectively, in the electronic density after electronic excitation).

In Vacuum	Planar Conformation	Non-Planar Conformation
Conformation	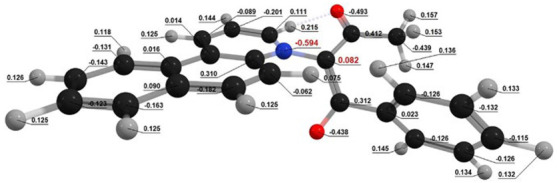 Q[-N-] = − 0.594 Q[-C-] = 0.082 *μ_e_* = 2.27 D	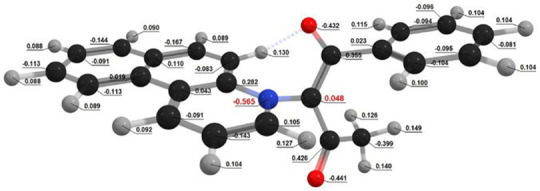 Q[-N-] = − 0.565 Q[-C-] = 0.048 *μ_e_* = 3.53 D
Hole–electron distribution	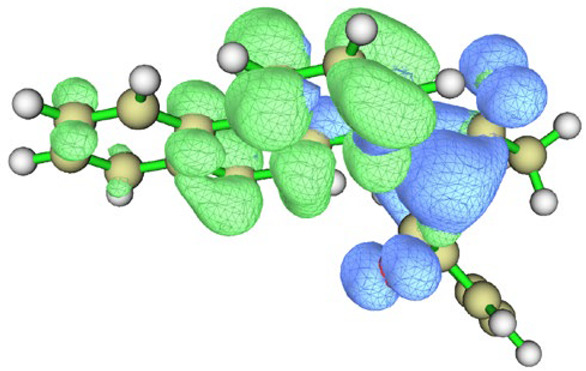	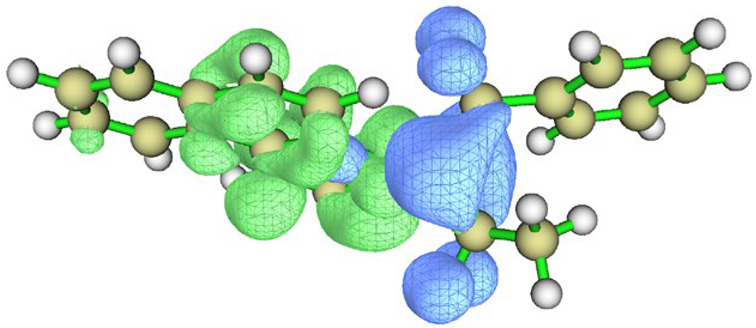
Charge density difference	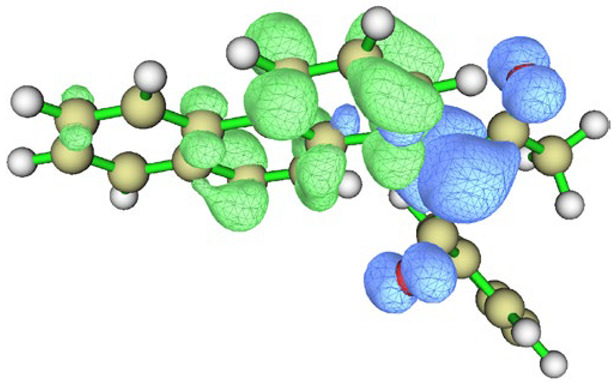	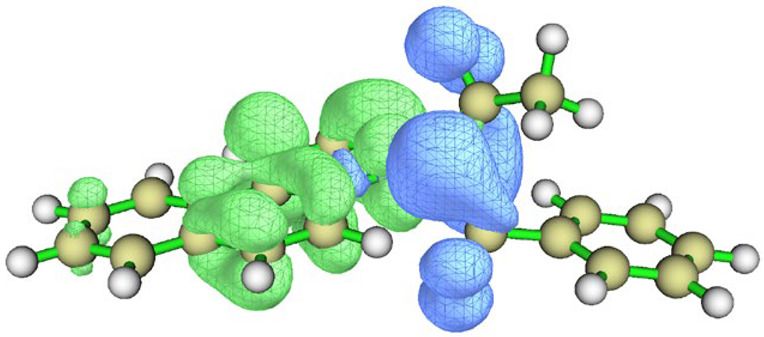
In CHX	Planar Conformation	Non-Planar Conformation
Conformation	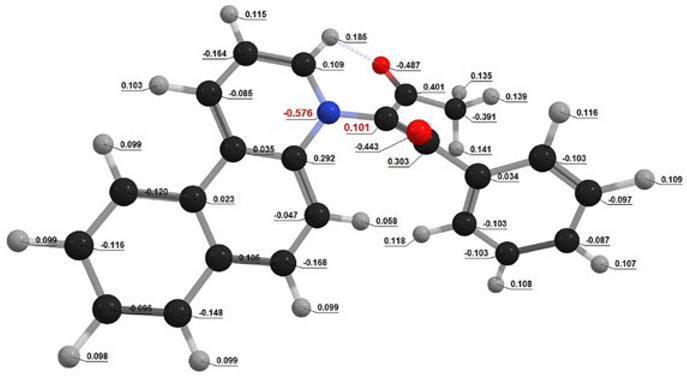 Q[-N-] = −0.576 Q[-C-] = 0.101 *μ_e_* = 2.77 D	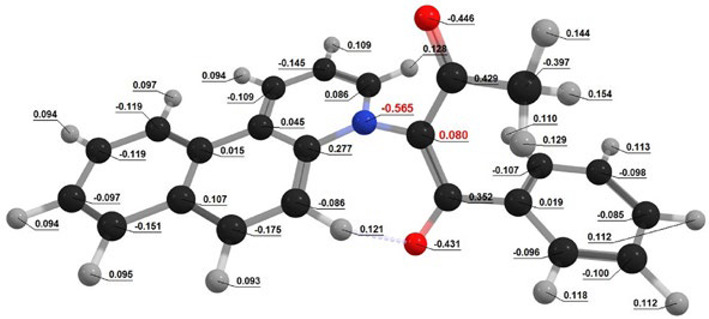 Q[-N-] = −0.565 Q[-C-] = 0.080 *μ_e_* = 4.87 D
Hole–electron distribution	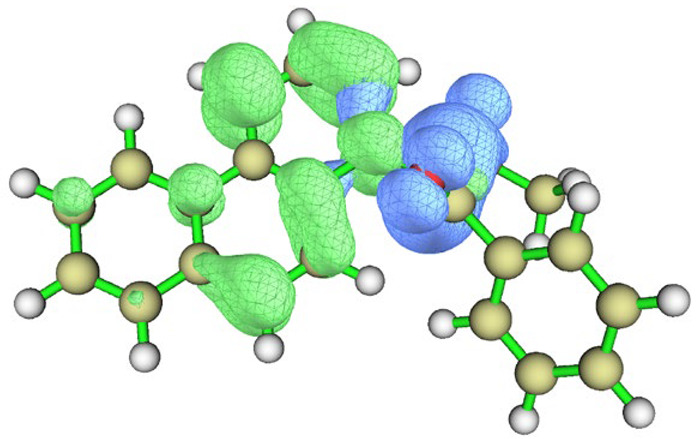	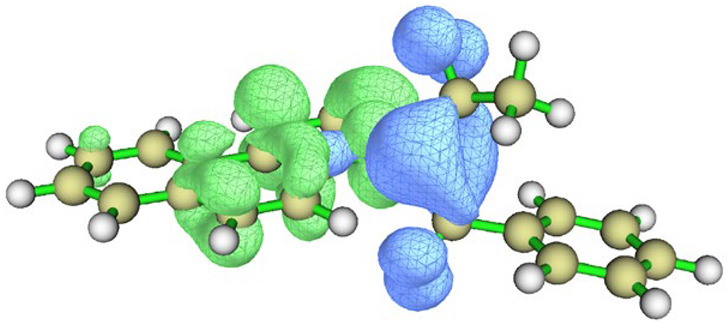
Charge density difference	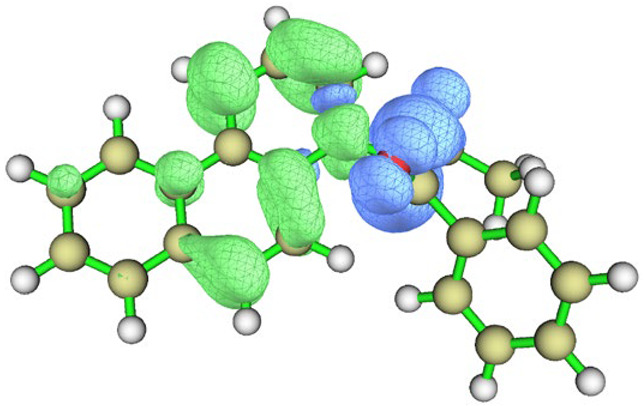	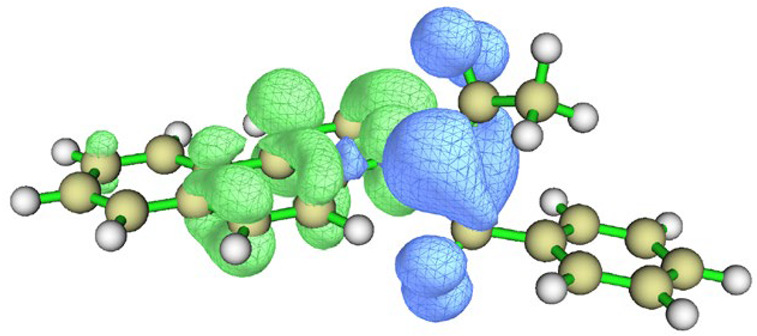
In Water	Planar Conformation	Non-Planar Conformation
Conformation	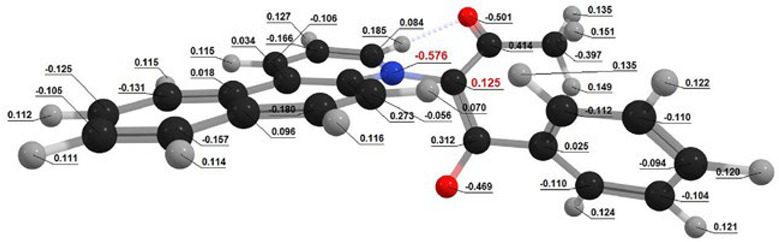 Q[-N-] = −0.576 Q[-C-] = 0.125 *μ_e_* = 4.15 D	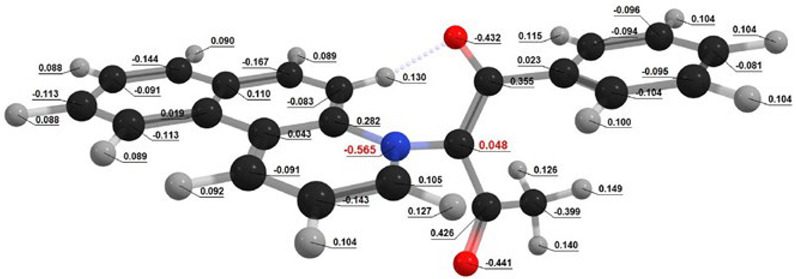 Q[-N-] = −0.565 Q[-C-] = 0.048 *μ_e_* = 6.09 D
Hole–electron distribution	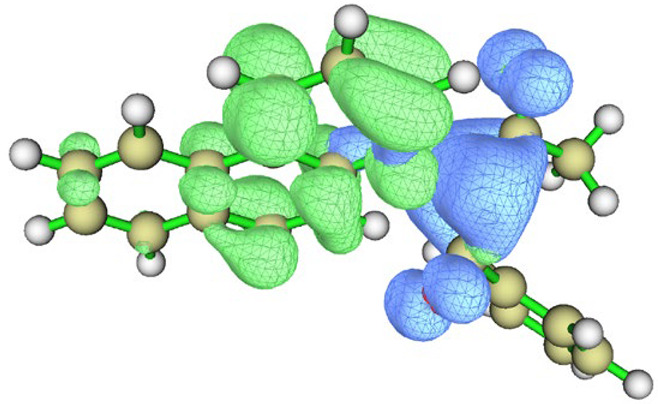	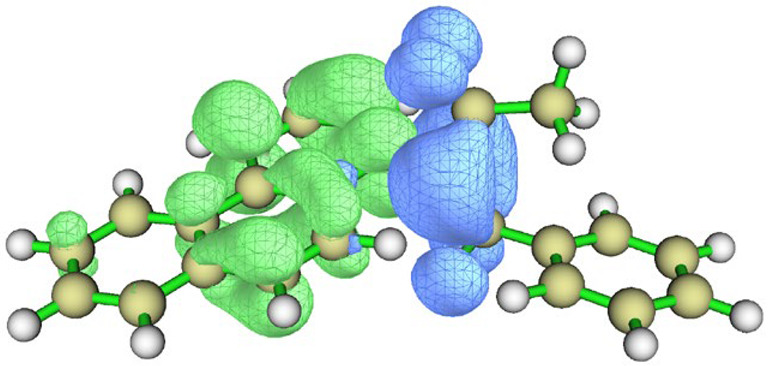
Charge density difference	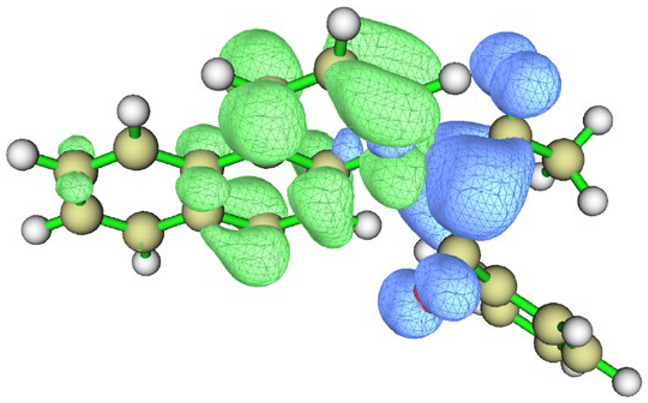	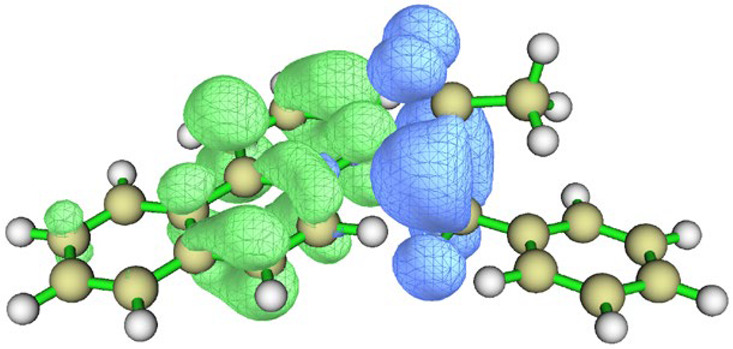

**Table 11:** The hole (blue) – electron (green) distribution during excitation and the charge density difference between states for the S_0_ → S_1_ transition (blue and green parts correspond to a decrease and an increase, respectively, in the electronic density after electronic excitation).

BfQ	Solvent	Hole–Electron Distribution	Charge Density Difference
**I2**	vacuum	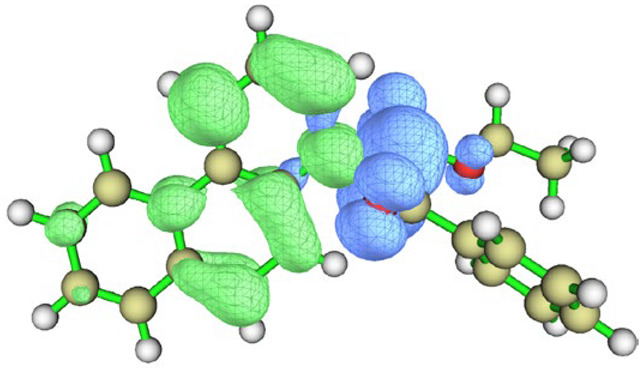	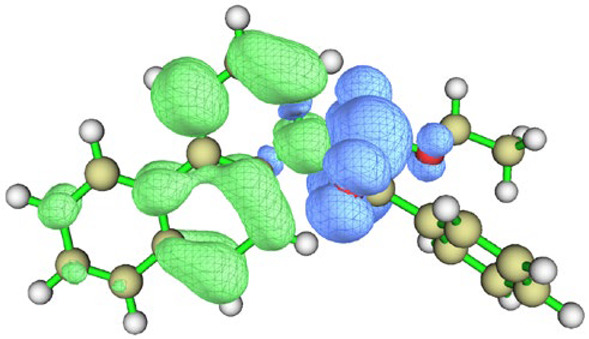
	cyclohexane	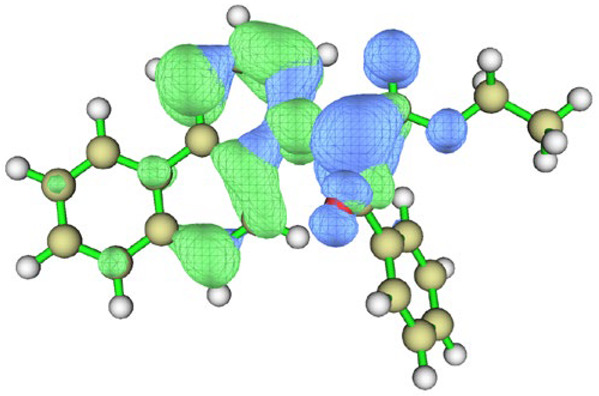	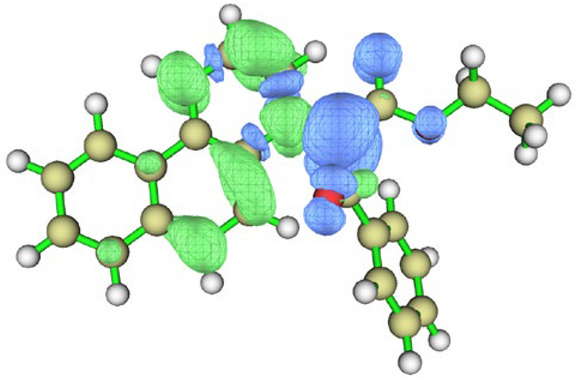
	water	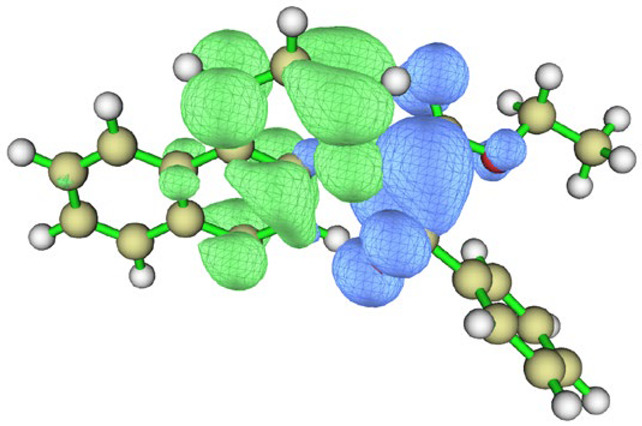	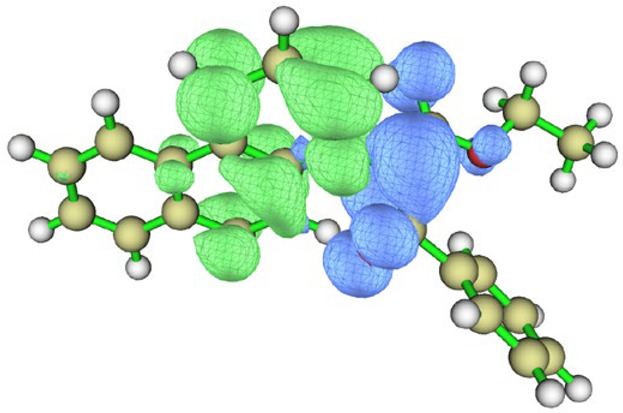
**I3**	vacuum	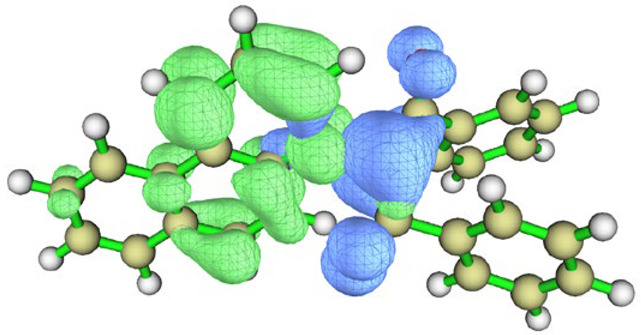	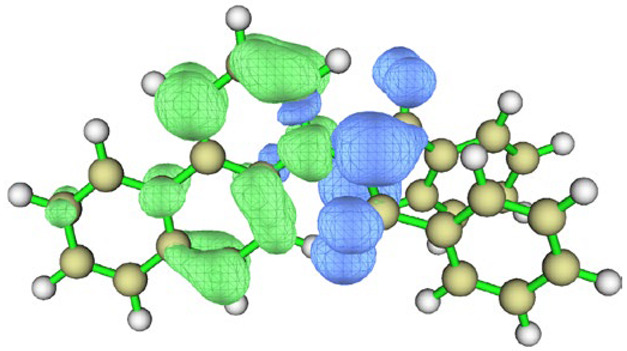
	cyclohexane	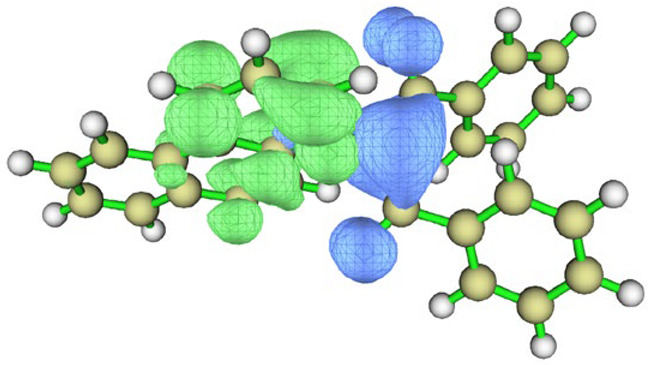	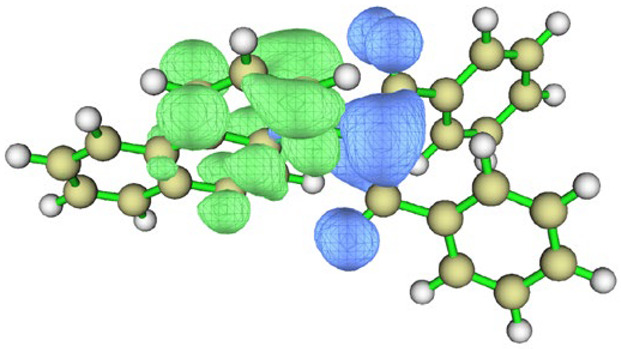
	water	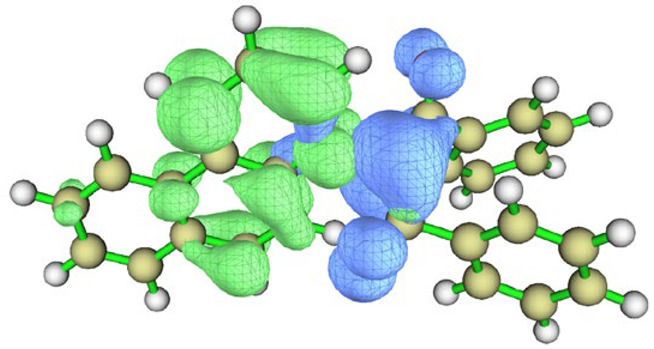	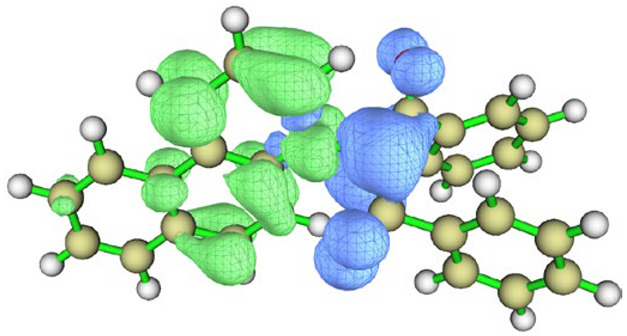

## Data Availability

The data are available by request.

## Supplementary Materials

The following supporting information can be downloaded at: https://www.mdpi.com/article/10.3390/molecules30153162/s1, Tables S1–S7: Multiparametric regression in the Kamlet–Taft approach. Calculated values of C_1μ_, C_1α_, and the R ratio. The Abe parameters (A, B, and C). Molecular descriptors of the solvents.
