# Supplementary material for: Solvatochromic and Computational Study of Three Benzo-[f]-Quinolinium Methylids with Photoinduced Charge Transfer
Source: Molecules. 2025 Jul 29;30(15):3162. doi: 10.3390/molecules30153162 (PMC12348346; doi:10.3390/molecules30153162)
Supplement: Supplementary file 1 [file molecules-30-03162-s001.zip › molecules-3737304-supplementary.pdf]

Supplementary Information

Solvatochromic and computational study of three benzo-[f]-quinolinium methylids with photoinduced charge transfer

Mihaela Iuliana Avadanei [1]*, Ovidiu Gabriel Avadanei [2] and Dana Ortansa Dorohoi [2,*]

[1] Petru Poni Institute of Macromolecular Chemistry, 41A Gr.Ghica Voda Alley, 700487, Iasi, Romania mavadanei@icmpp.ro
[2] Alexandru Ioan Cuza University, Faculty of Physics, 11 Carol I Blvd, 700506, Iasi, Romania. minu@uaic.ro
* Correspondence: ddorohoi@uaic.ro ; Tel.: (+ 40 749 641 954); mavadanei@icmpp.ro

Table S1. Multiparameter regression analysis applied to the solvatochromic parameters in the Kamlet – Taft approach

BFQ	K-T coefficient	Parameters	Standard error	t-Statistic	P (2-tail)
**I1**	ν_0	21079.48	181.76	116.0	<0.0001
	π^*	922.02	54.02	3.630	0.0009
	α	1728.32	236.58	7.305	<0.0001
	β	622.60	87.87	2.022	0.0513
	Coefficient of multiple correlations = 0.92		F = 58	N = 48	
**I2**	ν_0	20359.89	244.70	83.20	<0.0001
	π^*	887.83	141.99	2.596	0.0140
	α	2260.64	318.50	7.098	<0.0001
	β	1677.93	314.48	4.048	0.0003
	Coefficient of multiple correlations = 0.92		F = 75	N = 48	
**I3**	ν_0	19872.56	237.382	83.72	<0.0001
	π^*	1220.10	231.763	3.678	0.0008
	α	2660.40	308.98	4.121	0.0002
	β	1656.91	402.09	8.610	<0.0001
	Coefficient of multiple correlations = 0.95		F = 101	N = 48	

Table S2. Calculated values of $C_{1\mu}$, $C_{1\alpha}$ and the R ratio for I1

φ (degree)	$\mathbf{10^{36} C_{1\mu}}$	$\mathbf{10^{36} C_{1\alpha}}$	**R**
0	6.23	0.3539	0.06
20	6.19	0.3834	0.06
40	6.07	0.5145	0.085
60	5.498	1.0629	0.193
75	2.78	4.2561	1.876
77	0.36	6.1369	17.007
79	- 3.80	10.267	-2.70
80	-7.80	15.214	-1.95

Table S3. Calculated values of $C_{1\mu}$, $C_{1\alpha}$ and the R ratio for I2

φ (degree)	$\mathbf{10^{36} C_{1\mu}}$	$\mathbf{10^{36} C_{1\alpha}}$	**R**
**0**	10.8	0.166	0.015
**20**	10.77	0.185	0.017
**40**	10.41	0.254	0.024
**60**	10.37	0.606	0.059
**75**	8.44	2.51	0.298
80	4.34	7.24	1.67
81	-1.42	12.28	-8.66
81.5	-7.96	18.76	-2.36

Table S4. Calculated values of $C_{1\mu}$, $C_{1\alpha}$ and the R ratio for I3

φ (degree)	$\mathbf{10^{36} C_{1\mu}}$	$\mathbf{10^{36} C_{1\alpha}}$	**R**
**0**	8.47	0.184	0.015
**20**	8.46	0.213	0.017
**40**	8.45	0.339	0.024
**60**	8.39	0.865	0.059
**75**	8.30	0.469	0.298
77	8.42	27.75	3.29
80	-4.93	13.45	-2.73
81	-19.36	194.29	-1.43

Table S5. Molecular descriptors of the solvents: dipole moment (μ), polarizability (α), ionization potential (I), molecular mass (M) and density (ρ)

Nr.	Solvent	μ(g) (D)	α(g) (Å^3)	I(g) (eV)	M(g/mol)	ρ(g/cm^3)
**1**	Dioxane	0	9.44	9.52	88.11	1.417
**2**	p-Xylene	0	14.35	8.52	106.17	0.862
**3**	Benzene	0	10.44	9.25	78.11	0.868
**4**	Carbon tetrachloride	0	10.5	9.72	153.82	1.594
**5**	n-Heptane	0	13.61	10.35	100.2	0.683
**6**	1,3,5-Trimethylbenzene	0	16.12	8.76	120.2	0.864
**7**	Toluene	0.38	12.4	8.72	92.14	0.867
**8**	o-Xylene	0.64	14.25	8.56	106.17	0.88
**9**	Trichloroethylene	0.8	9.75	9.45	131.4	1.46
**10**	Chloroform	1.15	8.23	11.5	119.38	1.446
**11**	Methoxybenzene	1.38	13.1	8.2	108.14	0.995
**12**	1,2-Dichloroetane	1.43	8.68	10.49	173.84	2.447
**13**	Cyclohexanol	1.46	11.94	10	100.16	0.962
**14**	Chlorobenzene	1.5	13	9.07	112.56	1.11
**15**	Dichloromethane	1.6	6.66	11.32	84.93	1.33
**16**	Hexan-1-ol	1.6	12.4	8.98	102.175	0.814
**17**	Butan-1-ol	1.66	8.88	9.99	74.12	0.81
**18**	Propan-2-ol	1.66	6.67	9.9	60.1	0.786
**19**	Methyl acetate	1.67	6.99	10.51	74.08	0.972
**20**	Phenylmethanol	1.67	11.89	8.26	108.14	1.044
**21**	Propan-1-ol	1.68	6.67	10.52	60.1	0.803
**22**	Octan-1-ol	1.68	16.1	9.8	130.227	0.827
**23**	Ethanol	1.69	5.06	10.7	46.07	0.789
**24**	Methanol	1.7	3.21	10.85	32.04	0.792
**25**	Pentanol	1.7	11.58	10.42	88.15	0.814
**26**	Propanoic acid	1.75	6.96	10.44	74.08	0.99
**27**	2-Methylpropan-1-ol	1.76	9.07	10.12	74.12	0.802
**28**	3-Methylbutyl acetate	1.77	15.18	9.9	130.18	0.884
**29**	Ethyl acetate	1.78	9.7	10.11	88.11	0.902
**30**	Butyl acetate	1.84	13.42	10	116.16	0.883
**31**	Water	1.85	1.5	12.59	18	1
**32**	Pyridine	2.2	2.41	9.34	79.1	0.978
**33**	Propane-1,2-diol	2.27	8.01	10	76.1	1.036
**34**	Propane-1,3- diol	2.53	6.5	10.42	76.1	1.06
**35**	Butan-2-one	2.76	8.28	9.54	72.11	0.805
**36**	Acetone	2.8	6.27	9.89	58.08	0.971

**37**	4-Hydroxy-4-methylpentan-2-one	3.24	12.4	9.6	116.16	0.938
**38**	Formamide	3.73	4.08	10.2	45.04	1.133
**39**	1-Phenylethanone	3.81	14.37	9.77	120.14	1.028
**40**	Dimethylformamide	3.86	7.91	9.12	73.94	0.944
**41**	Acetonitrile	3.92	4.3	12.2	41.05	0.786
**42**	Dimethylsulfoxide	4.1	8	9.1	78.13	1.1
**43**	n-Hexane	0.09	10.6	10.18	86.18	0.659
**44**	1,2-Dibromoethane	1.04	10.69	9.45	187.862	2.18
**45**	Cyclohexanone	3.06	11.061	9.14	98.14	0.942
**46**	Pentane-2,4-dione	3.03	9.535	4.43	100.13	0.98
**47**	Ethane-1,2-diol	0	5.71	10.16	62.07	1.11
**48**	Propane-1,2,3-triol	2.56	7.86	11.06	92.09	1.261

Table S6. Values of the Abe parameter C for I1, I2 and I3

Nr.	Solvent	I2	I3	I4
1	Dioxane	1.598	1.506	1.415
2	p-Xylene	0.665	0.630	0.595
3	Benzene	1.001	0.945	0.891
4	Carbon tetrachloride	0.914	0.864	0.815
5	n-Heptane	0.528	0.500	0.473
6	1,3,5-Trimethylbenzene	0.566	0.537	0.507
7	Toluene	0.807	0.763	0.720
8	o-Xylene	0.684	0.647	0.611
9	Trichloroethylene	1.000	0.945	0.890
10	Chloroform	1.117	1.055	0.993
11	Methoxybenzene	0.784	0.742	0.700
12	1,2-Dichloroetane	1.352	1.275	1.199
13	Cyclohexanol	0.829	0.784	0.739
14	Chlorobenzene	0.858	0.811	0.765
15	Dichloromethane	1.546	1.457	1.369
16	Hexan-1-ol	0.649	0.615	0.581
17	Butan-1-ol	0.979	0.925	0.872
18	Propan-2-ol	1.232	1.163	1.094
19	Methyl acetate	1.237	1.167	1.098
20	Phenylmethanol	0.834	0.789	0.744
21	Propan-1-ol	1.266	1.194	1.124
22	Octan-1-ol	0.480	0.455	0.431
23	Ethanol	1.729	1.628	1.529
24	Methanol	2.713	2.548	2.386

25	Pentanol	0.787	0.745	0.703
26	Propanoic acid	1.266	1.195	1.124
27	2-Methylpropan-1-ol	0.967	0.914	0.861
28	3-Methylbutyl acetate	0.525	0.498	0.471
29	Ethyl acetate	0.900	0.851	0.802
30	Butyl acetate	0.610	0.578	0.546
31	Water	7.034	6.573	6.122
32	Pyridine	1.147	1.083	1.019
33	Propane-1,2-diol	1.296	1.223	1.150
34	Propane-1,3- diol	1.334	1.259	1.184
35	Butan-2-one	1.007	0.951	0.896
36	Acetone	1.678	1.580	1.484
37	4-Hydroxy-4-methylpentan-2-one	0.661	0.626	0.591
38	Formamide	2.771	2.602	2.437
39	1-Phenylethanone	0.713	0.675	0.637
40	Dimethylformamide	1.195	1.128	1.061
41	Acetonitrile	1.985	1.868	1.753
42	Dimethylsulfoxide	1.353	1.276	1.199
43	n-Hexane	0.615	0.582	0.550
44	1,2-Dibromoethane	1.058	0.999	0.941
45	Cyclohexanone	0.828	0.783	0.739
46	Pentane-2,4-dione	0.849	0.803	0.757
47	Ethane-1,2-diol	1.824	1.718	1.612
48	Propane-1,2,3-triol	1.306	1.232	1.1590

Table S7. Values of the Abe parameters A and B for I1, I2 and I3

Nr.	Solvent	$A \times 10^{12}$, erg			$B \times 10^{36}$, erg . cm^3		
		I1	I2	I3	I1	I2	I3
1	Dioxane	8.09E+00	8.09E+00	8.12E+00	1.85E+02	2.16E+02	9.11E+01
2	p-Xylene	7.77E+00	7.77E+00	7.79E+00	-1.14E+02	2.28E+02	1.05E+01
3	Benzene	8.01E+00	8.01E+00	8.03E+00	-4.76E+01	2.37E+02	1.86E+01
4	Carbon tetrachloride	8.15E+00	8.15E+00	8.18E+00	9.88E+01	1.45E+02	9.39E+01
5	n-Heptane	8.33E+00	8.33E+00	8.36E+00	7.09E+01	9.40E+01	6.91E+01
6	1,3,5-Trimethylbenzene	7.85E+00	7.85E+00	7.88E+00	-1.26E+02	2.36E+02	-3.18E+01
7	Toluene	6.50E+00	6.50E+00	6.52E+00	-2.57E+01	2.47E+02	3.51E+01
8	o-Xylene	5.16E+00	5.16E+00	5.18E+00	-2.79E+01	1.81E+02	4.15E+01
9	Trichloroethylene	3.74E+00	3.74E+00	3.75E+00	5.71E+00	8.84E+01	-7.39E+00
10	Chloroform	2.24E+00	2.24E+00	2.25E+00	1.06E+01	4.26E+01	-6.07E+01

**11**	Methoxybenzene	2.14E+00	2.14E+00	2.15E+00	-1.21E+01	3.01E+01	-2.21E+01
**12**	1,2-Dichloroetane	1.62E+00	1.62E+00	1.62E+00	1.01E+01	2.25E+01	-1.15E+01
**13**	Cyclohexanol	1.98E+00	1.98E+00	1.99E+00	-5.98E+01	-1.11E+02	-1.67E+02
**14**	Chlorobenzene	1.96E+00	1.96E+00	1.96E+00	-6.88E+00	2.46E+01	-2.60E+00
**15**	Dichloromethane	1.10E+00	1.10E+00	1.10E+00	3.39E+00	4.17E+00	-1.05E+01
**16**	Hexan-1-ol	1.70E+00	1.70E+00	1.71E+00	-8.10E+01	-8.28E+01	-1.81E+02
**17**	Butan-1-ol	1.27E+00	1.27E+00	1.27E+00	-6.91E+01	-9.17E+01	-1.37E+02
**18**	Propan-2-ol	9.88E-01	9.88E-01	9.91E-01	-6.16E+01	-7.08E+01	-1.17E+02
**19**	Methyl acetate	1.04E+00	1.04E+00	1.04E+00	-4.92E+00	3.90E+00	-2.75E+01
**20**	Phenylmethanol	1.49E+00	1.49E+00	1.50E+00	-5.28E+01	-9.57E+01	-1.52E+02
**21**	Propan-1-ol	9.87E-01	9.87E-01	9.90E-01	-5.45E+01	-7.86E+01	-1.21E+02
**22**	Octan-1-ol	1.99E+00	1.99E+00	2.00E+00	-9.05E+01	-1.38E+02	-1.88E+02
**23**	Ethanol	7.67E-01	7.67E-01	7.69E-01	-4.04E+01	-5.47E+01	-8.65E+01
**24**	Methanol	5.00E-01	5.00E-01	5.01E-01	-3.09E+01	-3.55E+01	-6.16E+01
**25**	Pentanol	1.54E+00	1.54E+00	1.55E+00	-5.95E+01	-1.03E+02	-1.52E+02
**26**	Propanoic acid	9.51E-01	9.51E-01	9.54E-01	-6.87E+01	-6.87E+01	-1.20E+02
**27**	2-Methylpropan-1-ol	1.17E+00	1.17E+00	1.18E+00	-6.14E+01	-8.70E+01	-1.18E+02
**28**	3-Methylbutyl acetate	1.76E+00	1.76E+00	1.77E+00	-3.97E+00	3.29E+00	-6.64E+01
**29**	Ethyl acetate	1.22E+00	1.22E+00	1.22E+00	-1.44E+01	6.90E+00	-1.60E+01
**30**	Butyl acetate	1.51E+00	1.51E+00	1.51E+00	-7.29E+00	1.78E+01	-6.08E+01
**31**	Water	2.14E-01	2.14E-01	2.15E-01	-1.00E+01	-1.11E+01	-2.00E+01
**32**	Pyridine	2.20E-01	2.20E-01	2.21E-01	-2.73E+01	-2.67E+01	-3.66E+01
**33**	Propane-1,2-diol	6.65E-01	6.65E-01	6.67E-01	-2.77E+01	-3.59E+01	-6.00E+01
**34**	Propane-1,3- diol	4.53E-01	4.53E-01	4.55E-01	-3.24E+01	-3.23E+01	-5.75E+01
**35**	Butan-2-one	4.69E-01	4.69E-01	4.70E-01	-7.26E+00	-5.89E+00	-1.78E+01
**36**	Acetone	3.55E-01	3.55E-01	3.56E-01	-8.56E-01	1.83E+00	-5.71E+00
**37**	4-Hydroxy-4-methylpentan-2-one	5.08E-01	5.08E-01	5.10E-01	-2.98E+01	-3.93E+01	-5.74E+01
**38**	Formamide	1.35E-01	1.35E-01	1.36E-01	-3.00E+00	-3.86E+00	-8.75E+00
**39**	1-Phenylethanone	4.33E-01	4.33E-01	4.34E-01	-6.62E-02	3.86E+00	-8.13E+00
**40**	Dimethylformamide	2.32E-01	2.32E-01	2.33E-01	-3.40E+00	-1.23E+00	-8.76E+00
**41**	Acetonitrile	1.37E-01	1.37E-01	1.37E-01	-2.58E-01	2.19E-01	-5.30E+00
**42**	Dimethylsulfoxide	2.09E-01	2.09E-01	2.09E-01	-6.19E-01	3.42E-01	-6.66E+00
**43**	n-Hexane	8.17E+00	8.17E+00	8.20E+00	3.47E+01	5.56E+01	-1.30E+01
**44**	1,2-Dibromoethane	2.89E+00	2.89E+00	2.90E+00	8.21E+00	6.38E+01	-1.26E+01
**45**	Cyclohexanone	4.99E-01	4.99E-01	5.01E-01	-4.61E+00	-4.01E+00	-1.35E+01
**46**	Pentane-2,4-dione	3.19E-01	3.19E-01	3.20E-01	-1.37E+01	-1.29E+01	-2.30E+01
**47**	Ethane-1,2-diol	8.28E+00	8.28E+00	8.30E+00	-4.91E+02	-5.31E+02	-9.11E+02
**48**	Propane-1,2,3-triol	5.41E-01	2.80E-07	5.42E-01	-3.06E+01	-3.41E+01	-5.80E+01
